# Rehabilitative Kurzzeitpflege – Optimierung der poststationären Versorgung von geriatrischen Patienten mit Rehabilitationsbedarf: Ergebnisse der REKUP-Studie

**DOI:** 10.1007/s00391-024-02367-4

**Published:** 2024-09-28

**Authors:** Anne Keilhauer, Christian Werner, Sandra Diekmann, Pauline zur Nieden, Kathrin Pahmeier, Anja Neumann, Anke Walendzik, Theresa Hüer, Pascal Raszke, Jürgen Wasem, Julia Frankenhauser-Mannuß, Norbert Specht-Leible, Jürgen M. Bauer

**Affiliations:** 1 Geriatrisches Zentrum, Universitätsklinikum Heidelberg, Agaplesion Bethanien Krankenhaus Heidelberg, Rohrbacher Straße 149, 69126 Heidelberg, Deutschland; 2 Essener Forschungsinstitut für Medizinmanagement (EsFoMed) GmbH, Bredeneyer Str. 2b, 45133 Essen, Deutschland; 3 Lehrstuhl für Medizinmanagement, Universität Duisburg-Essen, Thea-Leymann-Str. 9, 45127 Essen, Deutschland; 4 Unternehmensbereich Versorgungsgestaltung, Geschäftsbereich Versorgungsinnovationen & sektorenübergreifende Versorgungslösungen, AOK Baden-Württemberg, Presselstraße 19, 70191 Stuttgart, Deutschland

**Keywords:** Rehabilitation, Pflegemaßnahmen, Gebrechliche ältere Erwachsene, Qualität der Gesundheitsversorgung, Sektorenübergreifende Zusammenarbeit, Rehabilitation, Nursing care, Frail older adults, Quality of healthcare, Intersectoral collaboration

## Abstract

**Hintergrund:**

Geriatrische Patient*innen in Kurzzeitpflege (KZP) mit Rehabilitationsbedarf nach einem Krankenhausaufenthalt (KA) nehmen selten stationäre Rehabilitation (SR) in Anspruch und werden häufig in die Dauerpflege (DP) übergeleitet. Dies weist auf Optimierungsmöglichkeiten in der KZP hin.

**Ziel der Arbeit:**

Überprüfung der Wirksamkeit der Rehabilitativen Kurzzeitpflege (REKUP) zur Verbesserung der Versorgung geriatrischer Patient*innen in KZP mit Rehabilitationsbedarf nach KA.

**Methoden:**

Die Studie wurde als nichtrandomisierte Interventionsstudie mit historischer Kontrollgruppe (KG) durchgeführt. Die Interventionsgruppe (IG: *n* = 49) erhielt REKUP (aktivierend-therapeutische Pflege, funktionell-rehabilitative Therapien, psychosoziale Angebote, medizinische Betreuung), die KG (*n* = 57) die Regelversorgung während des KZP-Aufenthalts. Primäre Zielkriterien waren: Überleitung in die SR, Häuslichkeit und DP, negativ verändertes Versorgungsetting, Pflegegrad und Mortalität bis 3 Monate nach der KZP. Sekundäre Zielkriterien waren funktionelle, motorische und psychische Variablen.

**Ergebnisse:**

Die Überleitungsrate in SR (82 % vs. 37 %) und Häuslichkeit (86 % vs. 65 %) war in der IG höher (*p* < 0,05) als in der KG. Der Personenanteil mit Inanspruchnahme von DP (12 % vs. 35 %) und negativ verändertem Versorgungssetting (35 % vs. 60 %) war in der IG geringer (*p* < 0,01) als in der KG. Barthel-Index, visuelle Analogskala der EQ-5D und Schmerzskala verbesserten sich (*p* < 0,05) in der IG, nicht jedoch in der KG.

**Diskussion:**

Als neues Versorgungskonzept der KZP fördert REKUP die Überleitung in die SR, reduziert die Inanspruchnahme von DP und verbessert die Chancen auf Rückkehr in die Häuslichkeit sowie auf höhere Selbstständigkeit bei geriatrischen Patient*innen mit Rehabilitationsbedarf nach einem KA.

**Zusatzmaterial online:**

Zusätzliche Informationen sind in der Online-Version dieses Artikels (10.1007/s00391-024-02367-4) enthalten.

## Abstract (en)

**Background:**

Geriatric patients in subacute inpatient care (SC) with rehabilitation needs after hospitalization seldom utilize rehabilitative services and are often transitioned to long-term care (LTC), suggesting that their care in SC can be optimized.

**Objective:**

To evaluate the effectiveness of rehabilitative subacute inpatient care (REKUP) in improving the care of geriatric patients in SC with rehabilitation needs after hospitalization.

**Methods:**

The study was conducted as a nonrandomized intervention trial with an historical control group (CG). The intervention group (IG: *n* = 49) received REKUP (activating therapeutic care, functional rehabilitative therapy, psychosocial services, medical care), while the CG (*n* = 57) received usual care during SC. Primary outcomes were transition to inpatient rehabilitation, home, and LTC, deteriorated care setting, care level, and mortality within 3 months after SC. Secondary outcomes were functional, motor and psychological variables.

**Results:**

The transition rate to inpatient rehabilitation (82% vs. 37%) and home (86% vs. 65%) was higher (*p* < 0.05) in the IG than in the CG. The proportion of persons utilizing LTC (12% vs. 35%) and with deteriorated care setting (35% vs. 60%) was lower (*p* < 0.01) in the IG than in the CG. The Barthel Index, visual analogue scale of the EQ-5D, and numerical pain scale improved (*p* < 0.05) during the SC stay in the IG but not in the CG.

**Discussion:**

REKUP as a new care model for SC promotes the transition to inpatient rehabilitation, reduces the utilization of LTC and improves the chances of returning home and achieving greater independence in geriatric patients with rehabilitation needs after hospitalization.

Fortbestehende Funktionsbeeinträchtigungen über den Krankenhausaufenthalt (KA) hinaus und sinkende Verweildauern führen zu einer Zunahme der Inanspruchnahme von Kurzzeitpflege (KZP) bei geriatrischen Patient*innen nach der Entlassung aus dem Krankenhaus. Ein Teil dieser Patient*innen hat Rehabilitationsbedarf und -potenzial, der jedoch kaum systematisch Beachtung findet. Stationäre Rehabilitation (SR) nach der KZP wird selten in Anspruch genommen, und es zeigt sich eine hohe Überleitungsquote in die Dauerpflege (DP). Rehabilitative Kurzzeitpflege (REKUP) zielt darauf ab, die Versorgung dieser Patient*innen zu optimieren.

## Hintergrund

Bei etwa 40 % geriatrischer Patient*innen nach einem KA bestehen bei KZP-Aufnahme trotz fehlender Rehabilitationsfähigkeit Rehabilitationsbedarfe mit tatsächlichen Besserungspotenzialen, deren Vernachlässigung von erheblicher Bedeutung für den weiteren Versorgungsverlauf sind [7, 8]. So werden Rehabilitationsmaßnahmen nach der KZP nur selten in Anspruch genommen, und es fehlen Konzepte für die intersektorale Versorgung zwischen KA und SR [6–8]. Diese Inkongruenz sowie die hohe Überleitungsquote in die DP legen nahe, dass die poststationäre Versorgung in der KZP optimiert werden muss [8, 9]. Es fehlen Gesetzesgrundlagen und einheitliche Standards, die klar die Steuerungsfunktion der KZP nach einem KA abbilden [4, 9]. Wissenschaftliche Erkenntnisse zu Gesundheitszustand und -entwicklung des Kollektivs sowie zur Art und Qualität der Versorgung in der KZP sind kaum vorhanden [4, 12]. Rehabilitative Versorgungsmöglichkeiten in der KZP umfassen Heilmittel (Physio‑/Ergotherapie, Logopädie) und mobile geriatrische Rehabilitation durch vertragsärztliche Verordnungen sowie privat finanzierte oder über spezielle Programme angebotene zusätzliche rehabilitative Maßnahmen (z. B. physikalische Maßnahmen, Gedächtnistraining, Kunsttherapie). Aktuelle Untersuchungen legen nahe, dass ein beachtlicher Teil (35 %) geriatrischer Patient*innen mit Rehabilitationsbedarf nach einem KA keine therapeutischen Anwendungen (z. B. Physio‑/Ergotherapie, Logopädie, sozialtherapeutische Beratung) in der KZP erhält; der Erhalt jedoch die Überleitungsquote in die SR und die Rückkehr in die Häuslichkeit begünstigt [8].

Vor diesem Hintergrund wurde von der AOK Baden-Württemberg (BW) in Kooperation mit dem Geriatrischen Zentrum am Universitätsklinikum Heidelberg im AGAPLESION BETHANIEN KRANKENHAUS HEIDELBERG (ABKH) das Projekt „REKUP – Rehabilitative Kurzzeitpflege im stationären Umfeld: Ein Versorgungskonzept für Versicherte mit und ohne vorbestehende Pflegebedürftigkeit“ initiiert. Ziel war es, die Versorgung geriatrischer Patient*innen mit Rehabilitationsbedarf und -potenzial, aber ohne aktuell bestehende Rehabilitationsfähigkeit, die nach einem KA in die KZP entlassen wurden, zu verbessern.

Als wesentliche Zielkriterien wurden dabei die Förderung der Überleitung in die SR und ins häusliche Umfeld, die Stabilisierung des funktionellen Status sowie die Vermeidung oder Verringerung von Pflegebedürftigkeit definiert. Es wurde angenommen, dass REKUP durch aktivierend-therapeutische Pflege, regelmäßige und früh ansetzende rehabilitative Therapiemaßnahmen, psychosoziale Angebote und kontinuierliche medizinische Betreuung zur Erreichung dieser Zielkriterien beiträgt. Das Teilprojekt zur gesundheitsökonomische Evaluation von REKUP, welches auf Basis von Abrechnungsdaten der AOK BW und unter Verwendung einer aus Sekundärdaten der AOK BW gebildeten Kontrollgruppe (KG) durchgeführt wurde, weist auf erste Vorteile von REKUP gegenüber der herkömmlichen KZP in Bezug auf die Überleitung in die SR, die Vermeidung von DP und das Versterben bei gleichen durchschnittlichen Kosten hin [5]. Ziel des vorliegenden Teilprojekts war es, die Wirksamkeit von REKUP auf Basis von Primärdaten einer historischen KG in Bezug auf die definierten Zielkriterien zu überprüfen.

## Methodik

### Studiendesign und Stichprobe

Dieses REKUP-Teilprojekt wurde als prospektive Interventionsstudie mit historischer KG durchgeführt. Die Zielkriterien wurden bei Aufnahme (T1) und Entlassung (T2) sowie 3 Monate nach der Entlassung (T3) aus der KZP erfasst. Die Teilnehmenden (TN) der KG mit wurden vom Februar 2019 bis zum Februar 2020 in 13 Pflegeeinrichtungen der Regionen Rhein-Neckar und Nordbaden rekrutiert. TN der Interventionsgruppe (IG) wurden mit Implementierung von REKUP an 2 Modellkliniken (ABKH, cts Sankt Rochus Kliniken Bad Schönborn) vom Oktober 2020 bis zum März 2022 eingeschlossen. Einschlusskriterien waren: geriatrische/r Patient*in (≥ 70 Jahre), Aufnahme in die KZP nach einem KA, versichert bei der AOK BW, Rehabilitationsbedarf und -potenzial, aber noch ohne bestehende Rehabilitationsfähigkeit (d. h. notwendige körperliche/psychische Belastbarkeit), Kooperationsfähigkeit und schriftliches Einverständnis. Ausschlusskriterien waren: fehlende Aussicht auf Herstellung ausreichender Belastbarkeit, fortgeschrittene Demenz und terminale Erkrankung. Für die IG und KG galten dieselben Ein- und Ausschlusskriterien, abgesehen davon, dass in die KG auch nicht-AOK-BW-versicherte Personen aufgenommen wurden. Das Kriterium „Rehabilitationsbedarf und -potenzial ohne bestehende Rehabilitationsfähigkeit“ wurde gemäß der Begutachtungsanleitung „Vorsorge und Rehabilitation“ des Spitzenverbands der gesetzlichen Krankenversicherung (GKV) (§ 282 Sozialgesetzbuch [SGB] V) [10] definiert als das Vorliegen von Rehabilitationsbedürftigkeit, die voraussichtliche Erreichbarkeit von Rehabilitationsfähigkeit mit alltagsrelevanten Rehabilitationszielen und eine positive Rehabilitationsprognose. Die Prüfung des Kriteriums erfolgte durch Pflegefachkräfte in den Einrichtungen, basierend auf ihrer pflegerischen Expertise.

### Intervention

REKUP war ein trägerübergreifendes Angebot der Kranken- und Pflegeversicherung, wobei Leistungen der stationären KZP (§ 42 SGB XI, § 39c SGB V) um rehabilitative Maßnahmen ergänzt wurden. REKUP wurde auf den Rehabilitationsstationen der beiden Modellkliniken von den dort etablierten multiprofessionellen Teams durchgeführt. Mit Aufnahme wurden auf Basis eines geriatrischen Assessments individuelle Behandlungsziele formuliert und ein Behandlungsplan erstellt. Im Rahmen wöchentlicher interdisziplinärer Teamsitzungen wurde die Rehabilitationsfähigkeit (Vorliegen körperlicher und psychischer Belastbarkeit zur aktiven Teilnahme an der Rehabilitation) gemäß den Kriterien der Begutachtungsanleitung „Vorsorge und Rehabilitation“ des GKV-Spitzenverbands (§ 282 SGB V) [10] überprüft. Die Versorgungsleistungen entsprachen denen der geriatrischen SR – jedoch in reduzierter Anzahl und Dauer entsprechend der (noch) verminderten Belastbarkeit. Funktionell-rehabilitative Therapien umfassten Physio‑/Ergotherapie und Logopädie und wurden an den 5 Werktagen mit max. 4 Therapieeinheiten/Tag und für je max. 15 min durchgeführt. Psychosoziale Angebote enthielten Patienten- und Angehörigenberatung, psychologische Gesprächsangebote und weitere problemorientierte Beratung (z. B. Kontinenzberatung). Vorgesehen war eine Verweildauer von 21 Tagen. Bei Herstellung der Rehabilitationsfähigkeit wurden ein Antrag auf die SR gestellt und die TN vor Ort, in der SR der Modellklinik, ohne zusätzliche Verlegung weiterbehandelt. Die Vergütung von REKUP erfolgte auf Basis eines Tagessatzes, orientiert an den Entgelten der geriatrischen SR. Neue Prozesse wurden nur im Bereich Patientenmanagement und Abrechnungswesen installiert.

### Kontrolle

Die KG erhielt während des Aufenthalts in der KZP die Regelversorgung („usual care“).

### Datenerhebung

Primäre Zielkriterien waren die Häufigkeit (jeweils Personenanzahl) der Überleitungen in die SR, der Rehospitalisierungen, der Überleitungen in das häusliche Umfeld, der Inanspruchnahme von DP, eines negativ veränderten Versorgungssettings (definiert als Umzug in die DP, betreutes Wohnen oder barrierefreie Wohnung, neu engagierter ambulanter Pflegedienst oder Einzug einer 24-h-Pflegekraft) und die Mortalität sowie die Rehospitalisierungsfälle (Gesamtzahl) und der Pflegegrad (PG) im Zeitraum bis T3. Diese Kriterien wurden auf anhand der Pflegedokumentation und standardisierter Befragungen der TN, Angehörigen und Pflegefachkräfte vor Ort (T2) oder telefonisch (T3) erfasst.

Als sekundäre Zielkriterien wurden zu T1, T2 (vor Ort) und T3 (telefonisch) Barthel-Index (BI), EQ-5D-5L-Index und visuelle Analogskala (VAS), 5‑stufige Likert-Skala zur Lebenszufriedenheit und numerische Schmerzskala (NRS) erhoben. Die Esslinger Transferskala wurde zu T1 und T2 durchgeführt; der Fragebogen zur Messung der Patientenzufriedenheit (ZUF-8) zu T2.

Folgende Versorgungsleistungen in der KZP wurden anhand der Pflegedokumentation ermittelt: Verweildauer, Therapieerhalt (Physio‑/Ergotherapie, Logopädie, psychosoziale und sonstige Maßnahmen; ja vs. nein) und Arztkontakt (ja vs. nein). Für die IG wurden zusätzlich die Therapieeinheiten in REKUP erhoben.

Alter, Geschlecht, Wohnsituation, Haupt‑/Nebendiagnosen, Medikamente, Inanspruchnahme von Pflegedienst vor dem KA, Sturzhistorie, Mini-Mental State Examination, Geriatrische Depressions-Skala, Body-Mass-Index, Mini Nutritional Assessment-Short Form und Clinical Frailty Scale wurden als Basischarakteristika zu T1 erfasst.

### Statistische Analyse

Deskriptive Statistiken wurden als Häufigkeiten (*n*) und Prozente (%), Mediane und Interquartilsbereiche (IQR) oder Mittelwerte (MW) und Standardabweichungen (SD) angegeben. Gruppenunterschiede wurden anhand *χ*^2^- bzw. Fisher-Exact-Tests, Mann-Whitney-U-Tests oder *t*-Tests für unabhängige Stichproben analysiert. Veränderungen wurden mittels 2‑faktoriellen Varianzanalysen (ANOVA) mit Messwiederholung analysiert (Faktoren: Gruppe [IG vs. KG] und Zeit [T1, T2; T3]). Im Falle von signifikanten Interaktionen (Gruppe⋅und Zeit) wurden Bonferroni-korrigierte Post-hoc-Tests für paarweise Vergleiche berechnet. Fehlende Werte wurden im Sinne des „Intention-to-treat“(ITT)-Prinzips und unter der „Missing-at-random“-Annahme mittels multipler Imputation ersetzt („predictive mean matching“; 20 Imputationen, 10 Iterationen). Das Imputationsmodell umfasste alle Basischarakteristika, die Gruppenzugehörigkeit (KG vs. IG) sowie die primären und sekundären Zielkriterien, erfasst zu T2 und T3. Die Ergebnisse der imputierten Datensätze wurden nach den Rubin-Regeln zusammengefasst [13]. Für die primären Zielkriterien wurden Complete-Case-Analysen als ergänzende Sensitivitätsanalysen durchgeführt. Für die statistischen Analysen wurden IBM SPSS Statistics (IBM Corp., Version 29.0, Armonk, NY, USA) verwendet. Ein 2‑seitiger *p*-Wert <0,05 wurde als Signifikanzniveau festgelegt.

## Ergebnisse

### Eigenschaften der Stichprobe

Es wurden 49 TN aus den beiden REKUP-Modellkliniken in die IG und 57 TN aus den regionalen Pflegeeinrichtungen in die KG eingeschlossen. Die Gesamtstichprobe umfasste 71 (67 %) Frauen, war im Durchschnitt 82,0 ± 6,2 Jahre alt und funktionell mittelschwer eingeschränkt (BI = 51,6 ± 20,0 Pkt.). Weitere Basischarakteristika sind im Zusatzmaterial (Supplement 1) aufgeführt. Es ergaben sich darin keine signifikanten Gruppenunterschiede mit der Ausnahme, dass die IG häufiger orthopädische/unfallchirurgische und weniger häufig neurologisch/psychiatrisch Diagnosen aufwies, mehr Medikamente nahm und funktionell eingeschränkter war als die IG (*p* < 0,001–0,042).

### Versorgungsleistungen in KZP

Die Verweildauer war in der IG signifikant kürzer als in der KG (19 ± 4 Tage vs. 28 ± 17 Tage, *p* < 0,001). Alle TN der IG wurden therapeutisch behandelt. Die IG wurde insgesamt signifikant häufiger therapiert (*p* < 0,001) und erhielt jeweils signifikant häufiger Physio‑/Ergotherapie sowie Logopädie, psychosoziale und sonstige Maßnahmen im Vergleich zur KG (*p* < 0,001). In der IG war Physiotherapie die am häufigsten durchgeführte Therapie (12,4 ± 4,5 Einheiten je TN) (Zusatzmaterial: Supplement 2).

### Primäre Zielkriterien

**Abb. 1:**
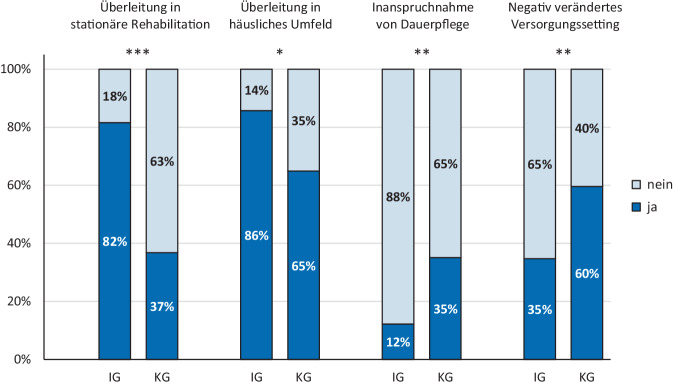
Prozentualer Anteil in der *IG* und *KG* mit Überleitung in die SR, Überleitung ins häusliche Umfeld bis T3, Inanspruchnahme von DP bis T3 und negativ verändertem Versorgungssetting zu T3. *p < 0,05, **p < 0,01, ***p < 0,001 (berechnet mit *χ*^2^-Tests)

Die Überleitungsquote in die SR nach KZP (82 % vs. 37 %, *p* < 0,001) und ins häusliche Umfeld bis T3 (86 % vs. 65 %, *p* = 0,021) war in der IG signifikant höher als in der KG. Zudem war der Anteil von TN mit Inanspruchnahme von DP bis T3 (12 % vs. 35 %, *p* = 0,006) und negativ verändertem Versorgungssetting zu T3 (35 % vs. 60 %, *p* = 0,008) in der IG signifikant geringer als in der KG (Abb. 1). Für die Rehospitalisierungsrate/-fälle, Mortalität und Zufriedenheit mit der Versorgung sowie die Veränderungen des PG ergaben sich keine signifikanten Gruppenunterschiede bzw. Wechselwirkungen (*p* = 0,319–0,999; Zusatzmaterial: Supplement 3). Die Complete-Case-Analyse bestätigt die Ergebnisse der ITT-Analyse (Zusatzmaterial: Supplement 4).

### Sekundäre Zielkriterien

Für den funktionellen Status, subjektiven Gesundheitszustand und Schmerz ergaben sich signifikante Interaktionseffekte (*p* = 0,007–0,040; Zusatzmaterial Supplement 5). Post-hoc-Tests zeigten, dass sich der BI, die EQ-5D VAS und die NRS von T1 bis T2 signifikant in der IG verbesserten (*p* < 0,001–0,030), nicht jedoch in der KG (*p* = 0,102–0,999). In der IG waren zudem der BI und die EQ-5D VAS zu T3 gegenüber T1 weiterhin signifikant verbessert (*p* < 0,001). In der KG waren keine solchen signifikanten Verbesserungen zu beobachten (BI: *p* = 0,198; EQ-5D VAS: *p* > 0,999).

Keine signifikanten Wechselwirkungen wurde für die Mobilität, Lebensqualität und Lebenszufriedenheit beobachtet (*p* = 0,159–0,466).

## Diskussion

In dieser Studie wurde untersucht, ob REKUP im Vergleich zur üblichen Versorgung bei geriatrischen Patient*innen in der KZP mit Rehabilitationsbedarf nach einem KA die Überleitung in die SR erhöht, die Rückkehr ins häusliche Umfeld fördert und die Inanspruchnahme von DP bzw. den Pflegebedarf reduziert.

Übergeordnetes Ziel von REKUP war es, die Versorgung an der Schnittstelle zwischen Akut- und Rehabilitationsbehandlung zu optimieren und besser an den individuellen Bedarf der TN auszurichten, um das volle Potenzial der TN auszuschöpfen und so eine Rückkehr ins häusliche Umfeld zu begünstigen sowie das Risiko für eine Rehospitalisierung oder eine DP zu verringern. Die Ergebnisse unterstreichen die Bedeutung der Herstellung der Rehabilitationsfähigkeit für den weiteren Verlauf der Selbstständigkeit der TN. Unabhängig von der Gruppenzugehörigkeit lag die Rückkehrquote ins häusliche Umfeld bei den TN, die in Rehabilitation übergleitet wurden, bei ≥90 % (IG: 36 von 40, 90 %; KG: 19 von 20, 95 %). Bei 82 % der TN konnte durch die REKUP die Rehabilitationsfähigkeit hergestellt werden. Die Überleitungsquote in die SR war in der IG gegenüber der KG signifikant erhöht und mehr als verdoppelt (82 % vs. 37 %). Bis T3 war der Anteil der Überleitungen in die eigene Häuslichkeit in der IG ebenfalls signifikant höher als in der KG, was auf eine erhöhte Selbstständigkeit und einen geringeren Pflegebedarf hinweist. Dieser positive Effekt von REKUP wird gestützt durch den signifikant geringeren Anteil der TN in der IG, die zu T3 ein negativ verändertes Versorgungssetting aufwiesen. Obwohl kein signifikanter Gruppenunterschied hinsichtlich der Entwicklung des PG zu beobachten war, untermauert die signifikante Verbesserung des BI in der IG über den Beobachtungszeitraum, die in der KG ausblieb, den Nutzen von REKUP gegenüber der herkömmlichen Versorgung während KZP auf Selbstständigkeit und Pflegebedürftigkeit.

Die Ergebnisse bestätigen die Annahme, dass die REKUP, verglichen mit der Regelversorgung, während des KZP-Aufenthalts bei geriatrischen Patient*innen mit Rehabilitationsbedarf nach einem KA den funktionellen Status stabilisiert, die Überleitung in die SR fördert und die Chancen auf die Rückkehr in die eigene Häuslichkeit sowie auf eine höhere Selbstständigkeit verbessert. Die REKUP fördert demnach die Umsetzung des Prinzips „Rehabilitation vor Pflege“ [14] und entspricht den vom Aktionsbündnis Kurzzeitpflege [1] als auch im Geriatriekonzept BW [11] geforderten konzeptionellen Weiterentwicklungen der KZP.

Die REKUP gewährleistet kontinuierlich therapeutische, ärztliche und psychosoziale Versorgung, was sich deutlich abhebt von der Regelversorgung in der KZP, bei der die Vorhaltung von Versorgungsleistungen nicht selten mehr Zufälligkeiten als Bedarfen folgt. Die Beobachtungen in der KG bestätigen die von anderen Studien beschriebene Inkongruenz zwischen Rehabilitationsbedarf geriatrischer Patient*innen in der KZP und der Inanspruchnahme rehabilitativer Leistungen während und nach der KZP [2, 7, 8]. Über ein Drittel (35 %) der KG waren therapeutisch unterversorgt. Dies mag u. a. auch durch den Bruch der ärztlichen Behandlungskontinuität in der KZP verursacht worden sein; fast ein Viertel (28 %) der KG hatte keinen Arztkontakt in der KZP. Im Gegensatz dazu erhielten alle TN der IG rehabilitative Therapiemaßnahmen und hatten Arztkontakt.

Grundsätzlich enthält die REKUP keine innovativen Elemente, sondern ist vielmehr eine „Light“-Variante der geriatrischen SR. Teamstrukturen und -verfahren sind bereits etabliert; neue Prozesse waren nur in den Bereichen Patientenmanagement und Abrechnungswesen erforderlich. Daher scheint die Implementierung von REKUP in andere geriatrische Rehabilitationskliniken grundsätzlich leicht möglich zu sein. Allerdings ist eine Umsetzung derzeit nur in Rehabilitationskliniken mit freien Kapazitäten realistisch. Bei Vollbelegung ist die Aufnahme eines Patient*innen in die SR attraktiver als die Aufnahme eines Patient*innen in die REKUP (v. a. aufgrund des Pflegeaufwands). Zusätzliche Kapazitäten für die REKUP vorzuhalten, ist für geriatrische Rehabilitationskliniken keine Option, solange die Höhe des tatsächlichen Bedarfs unklar ist und keine verbindlichen Zusagen der Entscheidungs- und Leistungsträger zu Förderung und Finanzierung der neuen Versorgungsform vorliegen.

Eine Möglichkeit zur Integration rehabilitativer Therapien in KZP-Einrichtungen außerhalb des stationären Umfelds könnte der Einsatz mobiler Rehabilitationsdienste sein, angelehnt an die mobile geriatrische Rehabilitation mit ihrem – vergleichbar zu REKUP (45 min/Tag, fraktioniert in max. 4 × 15-min Therapieeinheiten an 5 Tagen) – reduzierten Therapieumfang/ihrer reduzierten Therapiefrequenz (mindestens 45 min/Tag bei durchschnittlich 5 Therapieeinheiten/Woche), die parallel zur KZP erbracht werden und die Überleitung in die SR begünstigen kann. Dies setzt jedoch ebenfalls eine konzeptionell entsprechend optimierte KZP voraus [3].

### Limitationen

Die Rekrutierung der IG wurde durch die massiven Einschränkungen der COVID-19-Pandemie stark beeinträchtigt, weshalb die angestrebte Fallzahl (*n* = 200) nicht erreicht werden konnte und sich die statistische Teststärke der Studie reduzierte. Das Einschlusskriterium „Rehabilitationsbedarf und -potenzial ohne bestehende Rehabilitationsfähigkeit“ wurde durch Pflegefachkräfte anhand ihrer pflegerischen Expertise geprüft und nicht durch ein komplexes gutachterliches und/oder ärztliches Entscheidungsverfahren. Eine systematische Dokumentation des konkreten Grundes für die noch nicht vorliegende Rehabilitationsfähigkeit wurde dabei im Einzelfall nicht vorgenommen. Die Ergebnisse basieren auf einem nichtrandomisierten Studiendesign mit KG, was das Risiko systematischer Unterschiede zwischen den Gruppen zu T1 erhöht. Die KG zeigte tatsächlich einen höheren BI, weniger Medikamente und andere Hauptdiagnosen, was den Vergleich verzerrt haben könnte. Da ein höherer funktioneller Status und eine geringere Multimorbidität die primären Zielkriterien (z. B. Überleitung in Rehabilitation, Rückkehr ins häusliche Umfeld) eher begünstigen, gehen wir davon aus, dass der positive Effekt von REKUP möglicherweise unterschätzt wurde und noch deutlicher ausgefallen wäre, wenn diese initialen Gruppenunterschiede nicht bestanden hätten. Nachgeordnete multiple logistische Regressionsanalysen, die die primären Zielkriterien mit signifikanten Gruppenunterschieden in den bivariaten *χ*^2^-Tests als abhängige Variable (Überleitung in Rehabilitation, Überleitung ins häusliche Umfeld, Inanspruchnahme von DP, negativ verändertes Versorgungssetting) sowie die Gruppe (IG vs. KG) als unabhängige Variable berücksichtigen und für den BI, die Anzahl der Medikamente und die Hauptdiagnosen kontrolliert wurden, bestätigen überwiegend den positiven Effekt von REKUP (Zusatzmaterial: Supplement 6). Aufgrund der Untersuchungslogistik und allgemeiner pandemiebedingter Schutzmaßnahmen wurden Zielkriterien z. T. mittels verschiedener Befragungsmethoden (vor Ort vs. telefonisch; persönliche vs. Proxy-Interviews) erfasst, was zu Verzerrungen in den Antworten geführt haben könnte. Die fehlende Verblindung bei den Erhebungen könnte ebenfalls die Ergebnisse verzerrt haben. Es wurden mehrere primäre Zielkriterien verwendet, was die Aussagekraft der Ergebnisse potenziell verringert und das Risiko für Fehlinterpretationen erhöht.

## Fazit und Ausblick

Die Studie zeigt, dass REKUP bei geriatrischen Patient*innen in KZP mit Rehabilitationsbedarf nach einem KA die Überleitung in die SR fördert, die Inanspruchnahme von DP reduziert und die Chancen auf die Rückkehr in die eigene Häuslichkeit sowie auf eine höhere Selbstständigkeit verbessert. Die Ergebnisse legen nahe, dass die zentrale „Weichenstellerfunktion“ der KZP für die weitere Versorgungskarriere durch die Integration rehabilitativer Therapien substanziell verbessert wird. Eine Implementierung von REKUP erscheint in geriatrischen Rehabilitationskliniken leicht realisierbar. Solitäre Bereiche in Pflegeeinrichtungen könnten weitere potenzielle Settings sein, wenn hierbei die erforderlichen multiprofessionellen, rehabilitativ-therapeutischen sowie strukturellen Voraussetzungen darstellbar sind. Unabdingbare Voraussetzung für die Realisierung von REKUP sind jedoch verbindliche Zusagen der Entscheidungs- und Leistungsträger zu Stärkung und Finanzierung der geriatrischen Rehabilitation innerhalb und außerhalb der KZP.

## Fazit für die Praxis

- Geriatrische Patient*innen in der KZP mit Rehabilitationsbedarf nach einem KA bleiben oft therapeutisch und ärztlich unterversorgt und werden nur selten in eine SR übergeleitet.
- Die Integration rehabilitativer Therapien in die KZP fördert bei dieser Personengruppe die Überleitung in die SR, reduziert die Inanspruchnahme von DP und verbessert die Chancen auf eine Rückkehr nach Hause und eine höhere Selbstständigkeit.
- Die neue Versorgungsform REKUP ist mit geringem Aufwand in bestehende geriatrische Rehabilitationskliniken implementierbar.

## Supplementary Information

Supplement 1: Basischarakteristika der TN

Supplement 2: Versorgungsleistungen in KZP

Supplement 3: Unterschiede in primären Zielkriterien zwischen IG und KG

Supplement 4: Ergebnisse der Complete-Case-Analyse für die primären Zielkriterien

Supplement 5: Veränderungen in den sekundären Zielkriterien

Supplement 6: Ergebnisse der nachgeordneten multiplen logistischen Regressionsanalysen

## Data Availability

Die erhobenen Datensätze können auf begründete Anfrage beim korrespondierenden Autor angefordert werden.
