# Supplementary material for: Rehabilitative subacute inpatient care—Optimizing posthospital care for geriatric patients with rehabilitation needs: results of the REKUP study
Source: Z Gerontol Geriatr. 2024 Sep 28;58(4):289–95. [Article in German] doi: 10.1007/s00391-024-02367-4 (PMC12238062; doi:10.1007/s00391-024-02367-4)
Supplement: Supplementary file 1 — Supplement 1: Basischarakteristika der TN [file 391_2024_2367_MOESM1_ESM.docx]

**Supplement 1: Basischarakteristika der TN**

**Tab. S1** Vergleich in den Basischarakteristika zwischen IG und KG.

| **Charakteristik** | **IG** (*n* = 49) | **KG** (*n* = 57) | ***p*** |
| --- | --- | --- | --- |
| Alter [Jahre] | 82,3 ± 6,6 | 81,7 ± 5,9 | 0,601^1^ |
| Frauen [Personen] | 35 (71,4) | 36 (63,2) | 0,367^2^ |
| Wohnsituation [Personen] |  |  | 0,968^2^ |
| Alleinlebend | 27 (55,1) | 30 (52,6) |  |
| Mit Partner/Angehörigen | 18 (36,7) | 22 (38,6) |  |
| Sonstiges (Betreutes Wohnen, 24-h PK) [*n*] | 4 (8,2) | 5 (8,8) |  |
| Hauptdiagnose [Personen] |  |  | 0,042^2^ |
| Unfallchirurgisch/orthopädisch | 33 (67,4) | 26 (45,6) |  |
| Internistisch/onkologisch | 13 (26,5) | 20 (35,1) |  |
| Neurologisch/psychiatrisch | 3 (6,1) | 11 (19,3) |  |
| Nebendiagnose [Anzahl] | 8,9 ± 6,0 | 7,8 ± 4,4 | 0,270^1^ |
| Medikamente [Anzahl] | 12,4 ± 4,3 | 9,4 ± 3,8 | <0,001^1^ |
| PG bei Aufnahme [Grad] | 2 [1-3] | 2 [0-3] | 0,509^3^ |
| Pflegedienst vor KA [Personen] | 20 (40,8) | 20 (35,1) | 0,595^2^ |
| Barthel-Index [Pkt.] | 47 ± 18 | 55 ± 21 | 0,037^1^ |
| Mini-Mental State Examination [Pkt.] | 22,6 ± 5,9 | 22,7 ± 5,8 | 0,913^1^ |
| Geriatrische Depressions-Skala [Pkt.] |  |  |  |
| Mini Nutritional Assessment-Short Form [Pkt.] | 6,6 ± 2,6 | 6,1 ± 3,0 | 0,389^1^ |
| Body Mass Index [kg/m^2^] | 26,0 ± 4,8 | 25,2 ± 6,1 | 0,449^1^ |
| Clinical Frailty Scale [Pkt.] | 6,7 ± 0,5 | 6,7 ± 0,7 | 0,805^1^ |
| Sturzhistorie in den letzten 12 Monaten [Personen] | 40 (81,6) | 43 (75,4) | 0,351^3^ |
| Deskriptive Daten sind angegeben als MW ± SD, Median [IQR] oder *n* (%). *P*-Werte für *t*-Tests für unabhängige Stichproben^1^, *χ*^2^-Tests^2^ oder Mann-Whitney-*U*-Test^3^. | | | |
