# Supplementary material for: Rehabilitative subacute inpatient care—Optimizing posthospital care for geriatric patients with rehabilitation needs: results of the REKUP study
Source: Z Gerontol Geriatr. 2024 Sep 28;58(4):289–95. [Article in German] doi: 10.1007/s00391-024-02367-4 (PMC12238062; doi:10.1007/s00391-024-02367-4)
Supplement: Supplementary file 2 — Supplement 2: Versorgungsleistungen in KZP [file 391_2024_2367_MOESM2_ESM.docx]

**Supplement 2: Versorgungsleistungen in KZP**

**Tab. S2** Versorgungsleistungen der IG und KG während des KZP-Aufenthalts.

| **Variable** | **IG** (*n*=49) | **KG** (*n*=57) | ***p*** |
| --- | --- | --- | --- |
| Verweildauer [Tage] | 18,8 ± 4,4 | 28,2 ± 17,7 | <0,001^1^ |
| Therapien erhalten [Personen] | 49 (100,0) | 37 (64,9) | <0,001^2^ |
| Physiotherapie |  |  |  |
| Erhalten [Personen] | 49 (100,0) | 35 (61,4) | <0,001^2^ |
| Einheiten [Anzahl] | 12,4 ± 4,5 | - |  |
| Ergotherapie |  |  |  |
| Erhalten [Personen] | 46 (93,9) | 1 (1,8) | <0,001^2^ |
| Einheiten [Anzahl] | 6,3 ± 3,1 | - |  |
| Logopädie |  |  |  |
| Erhalten [Personen] | 11 (22,4) | 1 (1,8) | <0,001^2^ |
| Einheiten [Anzahl] | 6,5 ± 5,7 | - |  |
| Psychosoziale Maßnahmen |  |  |  |
| Erhalten [Personen] | 22 (44,9) | 0 (0) | <0,001^2^ |
| Einheiten [Anzahl] | 3,5 ± 2,9 | - |  |
| Sonstige Maßnahmen [Personen]^a^ |  |  |  |
| Erhalten [Personen] | 46 (93,9) | 8 (14,0) | <0,001^2^ |
| Einheiten [Anzahl] | 8,0 ± 6,2 | - |  |
| Arztkontakt [Personen] | 49 (100,0) | 41 (71,9) | <0,001^2^ |
| Deskriptive Daten angegeben als MW±SD oder *n*(%). *P*-Werte für *t*-Test für unabhängige Stichproben^1^ oder *χ*^2^-Tests bzw. Fisher-Exact-Tests^2^. ^a^Sonstige Maßnahmen: psychologische Diagnostik, supportive Einzelgespräche, zusätzliche Funktionstrainings, problemorientierte Beratung (z.B. Kontinenzberatung), Gruppentherapie (z.B. Balance-Gruppe), physikalische Anwendungen, Kunst und Werken, Angehörigenberatung. | | | |
