# Supplementary material for: Rehabilitative subacute inpatient care—Optimizing posthospital care for geriatric patients with rehabilitation needs: results of the REKUP study
Source: Z Gerontol Geriatr. 2024 Sep 28;58(4):289–95. [Article in German] doi: 10.1007/s00391-024-02367-4 (PMC12238062; doi:10.1007/s00391-024-02367-4)
Supplement: Supplementary file 3 — Supplement 3: Unterschiede in primären Zielkriterien zwischen IG und KG [file 391_2024_2367_MOESM3_ESM.docx]

**Supplement 3: Unterschiede in primären Zielkriterien zwischen IG und KG**

**Tab. S3** Gruppenunterschiede in den primären Zielkriterien

| **Primäre Zielkriterien** | **IG** (*n*=49) | **KG** (*n*=57) | ***p*** |
| --- | --- | --- | --- |
| Überleitung in SR nach KZP [Personen] | 40(81,6) | 21(36,8) | <0,001^1^ |
| Rehospitalisierung bis T2 [Personen] | 11(22,4) | 9(15,8) | 0,331^1^ |
| Rehospitalisierung bis T3 [Personen] | 24(49,0) | 24(42,1) | 0,466^1^ |
| Rehospitalisierungsfälle bis T2 [Anzahl] | 0[0-0] | 0[0-0] | 0,332^2^ |
| Rehospitalisierungsfälle bis T3 [Anzahl] | 0[0-1] | 0[0-1] | 0,319^2^ |
| Überleitung ins häusliche Umfeld bis T3 [Personen] | 42(85,7) | 37(64,9) | 0,021^1^ |
| Inanspruchnahme von DP bis T3 [Personen] | 6(12,2) | 20(35,1) | 0,006^1^ |
| Negativ verändertes Versorgungssetting zu T3 [Personen] | 17(34,7) | 34(59,6) | 0,008^1^ |
| Verstorben bis T2 [Personen] | 0(0,0) | 1(1,8) | >0,999^1^ |
| Verstorben bis T3 [Personen] | 7(14,3) | 6(10,5) | 0,570^1^ |
| Zufriedenheit mit Versorgung (ZUF-8) [Pkt.] | 22,9±7,3 | 22,7±6,3 | 0,868^3^ |
| Deskriptive Daten angegeben als *n*(%), Median[IQR] oder MW±SD. *P*-Werte für *χ*^2^-Tests bzw. Fisher-Exact-Tests^1^, Mann-Whitney-*U*-Tests^2^ oder *t*-Test für unabhängige Stichproben^3^. | | | |

**Tab. S4** Entwicklung des PG in der IG und KG über den Beobachtungszeitraum

| **PG** | **T1** | **T2** | **T3** | ***p*** |
| --- | --- | --- | --- | --- |
| IG (*n*=49) | 1,6±1,3 | 1,8±1,3 | 2,3±1,1 | 0,494 |
| KG (*n*=57) | 1,8±1,1 | 2,1±1,1 | 2,4±1,2 |  |
| Deskriptive Daten angegeben als MW±SD. *P*-Wert für Interaktionseffekt (Gruppe×Zeit) einer ANOVA mit Messwiederholung. | | | | |
