# Supplementary material for: Rehabilitative subacute inpatient care—Optimizing posthospital care for geriatric patients with rehabilitation needs: results of the REKUP study
Source: Z Gerontol Geriatr. 2024 Sep 28;58(4):289–95. [Article in German] doi: 10.1007/s00391-024-02367-4 (PMC12238062; doi:10.1007/s00391-024-02367-4)
Supplement: Supplementary file 4 — Supplement 4: Ergebnisse der Complete-Case-Analyse für die primären Zielkriterien [file 391_2024_2367_MOESM4_ESM.docx]

**Supplement 4: Ergebnisse der Complete-Case-Analyse für die primären Zielkriterien**

**Tab. S5** Gruppenunterschiede in den primären Zielkriterien.

| **Primäre Zielkriterien** | **IG** | | **KG** | | ***p*** |
| --- | --- | --- | --- | --- | --- |
|  | ***n*** | **Deskriptive**  **Statistik** | ***n*** | **Deskriptive**  **Statistik** |  |
| Überleitung in SR nach KZP [Personen] | 48 | 40 (83,3) | 50 | 21 (42,0) | <0,001^1^ |
| Rehospitalisierung bis T2 [Personen] | 49 | 11 (22,4) | 56 | 8 (14,3) | 0,278^1^ |
| Rehospitalisierung bis T3 [Personen] | 43 | 21 (48,8) | 53 | 22 (41,5) | 0,473^1^ |
| Rehospitalisierungsfälle bis T2 [Anzahl] | 49 | 0 [0-0] | 56 | 0 [0-0] | 0,281^2^ |
| Rehospitalisierungsfälle bis T3 [Anzahl] | 39 | 0 [0-1] | 52 | 0 [0-1] | 0,520^2^ |
| Überleitung ins häusliche Umfeld bis T3 [Personen] | 45 | 40 (88,9) | 52 | 35 (67,3) | 0,011^1^ |
| Inanspruchnahme von Dauerpflege bis T3 [Personen] | 40 | 4 (10,0) | 55 | 17 (30,9) | 0,015^1^ |
| Negativ verändertes Versorgungssetting zu T3 [Personen] | 40 | 12 (30,0) | 51 | 31 (60,8) | 0,004^1^ |
| Verstorben bis T2 [Personen] | 49 | 0 (0,0) | 57 | 1 (1,8) | >0,999^1^ |
| Verstorben bis T3 [Personen] | 41 | 3 (7,3) | 57 | 6 (10,5) | 0,587^1^ |
| Zufriedenheit mit Versorgung (ZUF-8) [Pkt.] | 33 | 23,6 ± 4,8 | 45 | 23,2 ± 5,5 | 0,763^3^ |
| Deskriptive Daten angegeben als *n* (%), Median [IQR] oder MW ± SD. *P*-Werte für *χ*^2^-Tests bzw. Fisher-Exact-Tests^1^, Mann-Whitney-*U*-Tests^2^ oder t-test für unabhängige Stichproben^3^. | | | | | |

**Tab. S6** Entwicklung des Pflegegrads in der IG und KG über den Beobachtungszeitraum.

| **Pflegegrad** | **T1** | **T2** | **T3** | ***p*** |
| --- | --- | --- | --- | --- |
| IG (*n* = 38) | 1,6 ± 1,3 | 1,8 ± 1,3 | 2,2 ± 1,1 | 0,175 |
| KG (*n* = 45) | 1,9 ± 1,0 | 2,1 ± 0,9 | 2,3 ± 1,0 |  |
| Deskriptive Daten angegeben als MW ± SD. *P*-Wert für Interaktionseffekt (Gruppe × Zeit) einer ANOVA mit Messwiederholung. | | | | |
