# Supplementary material for: Rehabilitative subacute inpatient care—Optimizing posthospital care for geriatric patients with rehabilitation needs: results of the REKUP study
Source: Z Gerontol Geriatr. 2024 Sep 28;58(4):289–95. [Article in German] doi: 10.1007/s00391-024-02367-4 (PMC12238062; doi:10.1007/s00391-024-02367-4)
Supplement: Supplementary file 5 — Supplement 5: Veränderungen in den sekundären Zielkriterien [file 391_2024_2367_MOESM5_ESM.docx]

**Supplement 5: Veränderungen in den sekundären Zielkriterien**

**Tab. S7** Veränderungen in den sekundären Zielkriterien in der IG und KG über den Beobachtungszeitraum.

| **Sekundäre Zielkriterien** | **T1** | **T2** | **T3** | ***p*-Wert** |
| --- | --- | --- | --- | --- |
| Barthel Index |  |  |  |  |
| IG (*n* = 49) | 47,2 ± 18,0 | 57,4 ± 20,3 | 64,7 ± 28,1 | 0,007 |
| KG (*n* = 57) | 55,4 ± 21,1 | 60,3 ± 24,4 | 61,2 ± 25,9 |  |
| Esslinger Transferskala |  |  |  |  |
| IG (*n* = 49) | 1,7 ± 1,5 | 1,2 ± 1,4 | - | 0,159 |
| KG (*n* = 57) | 1,2 ± 1,7 | 1,1 ± 1,6 | - |  |
| EQ-5D-5L Index |  |  |  |  |
| IG (*n* = 49) | 0,41 ± 0,32 | 0,56 ± 0,29 | 0,52 ± 0,46 | 0,466 |
| KG (*n* = 57) | 0,47 ± 0,30 | 0,57 ± 0,30 | 0,53 ± 0,48 |  |
| EQ-5D-5L visuelle Analogskala |  |  |  |  |
| IG (*n* = 49) | 41,9 ± 24,2 | 55,0 ± 27,4 | 55,8 ± 11,3 | 0,029 |
| KG (*n* = 57) | 51,9 ± 20,6 | 57,1 ± 23,5 | 54,4 ± 15,5 |  |
| Lebenszufriedenheit |  |  |  |  |
| IG (*n* = 49) | 3,0 ± 1,4 | 3,1 ± 1,2 | 3,3 ± 1,4 | 0,380 |
| KG (*n* = 57) | 3,1 ± 1,1 | 2,9 ± 1,3 | 3,2 ± 1,3 |  |
| Schmerz |  |  |  |  |
| IG (*n* = 49) | 5,0 ± 3,2 | 3,8 ± 2,9 | 4,3 ± 2,1 | 0,040 |
| KG (*n* = 57) | 3,2 ± 2,9 | 3,4 ± 3,2 | 3,7 ± 2,4 |  |
| Deskriptive Daten angegeben als MW ± SD. *P*-Werte für Interaktionseffekt (Gruppe × Zeit) der ANOVAs mit Messwiederholung. | | | | |
