# Supplementary material for: Rehabilitative subacute inpatient care—Optimizing posthospital care for geriatric patients with rehabilitation needs: results of the REKUP study
Source: Z Gerontol Geriatr. 2024 Sep 28;58(4):289–95. [Article in German] doi: 10.1007/s00391-024-02367-4 (PMC12238062; doi:10.1007/s00391-024-02367-4)
Supplement: Supplementary file 6 — Supplement 6: Ergebnisse der nachgeordneten multiplen logistischen Regressionsanalysen [file 391_2024_2367_MOESM6_ESM.docx]

**Supplement 6: Ergebnisse der nachgeordneten multiplen logistischen Regressionsanalysen**

**Tab. S8** Multiple logistische Regression für die Überleitung in stationäre Rehabilitation.

| **Variable** | **B** | **SE** | ***p*** | **OR (95% KI)** |
| --- | --- | --- | --- | --- |
| Gruppe | 1,690 | 0,530 | 0,001 | 5,42 (1,92-15,32) |
| Barthel-Index | 0,003 | 0,012 | 0,802 | 1,00 (0,98-1,03) |
| Medikamente | 0,014 | 0,064 | 0,828 | 1,01 (0,90-1,15) |
| Hauptdiagnose |  |  |  |  |
| Unfallchirurgisch/orthopädisch (Ref.) | - | - | - | - |
| Internistisch/onkologisch | -0,316 | 0,530 | 0,551 | 0,73 (0,26-2,06) |
| Neurologisch/psychiatrisch | -0,810 | 0,717 | 0,259 | 0,45 (0,11-1,82) |
| Kodierungen: Überleitung in stationäre Rehabilitation: 0 = nein, 1 = ja; Gruppe: 0 = KG, 1 = IG; Hauptdiagnose: 0 = nein, 1 = ja; Barthel-Index und Medikamente wurden metrisch in das Modell aufgenommen.  Abkürzungen: B = Regressionskoeffizient, SE = Standardfehler, OR = Odds Ratio, KI = Konfidenzintervall, Ref. = Referenzkategorie. | | | | |

**Tab. S9** Multiple logistische Regression für die Überleitung ins häusliche Umfeld.

| **Variable** | **B** | **SE** | ***p*** | **Odds Ratio (95% KI)** |
| --- | --- | --- | --- | --- |
| Gruppe | 1,205 | 0,590 | 0,041 | 3,34 (1,05-10,62) |
| Barthel-Index | 0,039 | 0,014 | 0,005 | 1,04 (1,01-1,07) |
| Medikamente | 0,060 | 0,078 | 0,441 | 1,06 (0,91-1,24) |
| Hauptdiagnose |  |  |  |  |
| Unfallchirurgisch/orthopädisch (Ref.) | - | - | - | - |
| Internistisch/onkologisch | -0,607 | 0,592 | 0,306 | 0,55 (0,17-1,74) |
| Neurologisch/psychiatrisch | -0,905 | 0,740 | 0,221 | 0,41 (0,10-1,73) |
| Kodierungen: Überleitung ins häusliche Umfeld: 0 = nein, 1 = ja; Gruppe: 0 = KG, 1 = IG; Hauptdiagnose: 0 = nein, 1 = ja; Barthel-Index und Medikamente wurden metrisch in das Modell aufgenommen.  Abkürzungen: B = Regressionskoeffizient, SE = Standardfehler, OR = Odds Ratio, KI = Konfidenzintervall, Ref. = Referenzkategorie. | | | | |

**Tab. S10** Multiple logistische Regression für die Inanspruchnahme von Dauerpflege.

| **Variable** | **B** | **SE** | ***p*** | **Odds Ratio (95% KI)** |
| --- | --- | --- | --- | --- |
| Gruppe | -1,267 | 0,611 | 0,038 | 0,28 (0,09-0,93) |
| Barthel-Index | -0,031 | 0,014 | 0,027 | 0,97 (0,94-1,00) |
| Medikamente | -0,101 | 0,073 | 0,166 | 0,90 (0,78-1,04) |
| Hauptdiagnose |  |  |  |  |
| Unfallchirurgisch/orthopädisch (Ref.) | - | - | - | - |
| Internistisch/onkologisch | 1,210 | 0,595 | 0,042 | 3,35 (1,04-10,76) |
| Neurologisch/psychiatrisch | 1,294 | 0,746 | 0,083 | 3,65 (0,85-15,75) |
| Kodierungen: Inanspruchnahme von Dauerpflege: 0 = nein, 1 = ja; Gruppe: 0 = KG, 1 = IG; Hauptdiagnose: 0 = nein, 1 = ja; Barthel-Index und Medikamente wurden metrisch in das Modell aufgenommen.  Abkürzungen: B = Regressionskoeffizient, SE = Standardfehler, OR = Odds Ratio, KI = Konfidenzintervall, Ref. = Referenzkategorie. | | | | |

**Tab. S11** Multiple logistische Regression für ein negativ verändertes Versorgungssetting.

| **Variable** | **B** | **SE** | ***p*** | **Odds Ratio (95% KI)** |
| --- | --- | --- | --- | --- |
| Gruppe | -0,950 | 0,495 | 0,055 | 0,39 (0,15-1,02) |
| Barthel-Index | -0,025 | 0,014 | 0,071 | 0,98 (0,95-1,00) |
| Medikamente | -0,075 | 0,080 | 0,351 | 0,93 (0,79-1,09) |
| Hauptdiagnose |  |  |  |  |
| Unfallchirurgisch/orthopädisch (Ref.) | - | - | - | - |
| Internistisch/onkologisch | 0,763 | 0,524 | 0,146 | 2,14 (0,77-6,00) |
| Neurologisch/psychiatrisch | 1,260 | 0,759 | 0,097 | 3,52 (0,80-15,63) |
| Kodierungen: Negative verändertes Versorgungssetting: 0 = nein, 1 = ja; Gruppe: 0 = KG, 1 = IG; Hauptdiagnose: 0 = nein, 1 = ja; Barthel-Index und Medikamente wurden metrisch in das Modell aufgenommen.  Abkürzungen: B = Regressionskoeffizient, SE = Standardfehler, OR = Odds Ratio, KI = Konfidenzintervall, Ref. = Referenzkategorie. | | | | |
